# Controlled alkali etching of MOFs with secondary building units for low-concentration CO_2_ capture†

**DOI:** 10.1039/d3sc03213b

**Published:** 2023-07-15

**Authors:** Hong Dong, Lihua Li, Can Li

**Affiliations:** a State Key Laboratory of Catalysis, Dalian National Laboratory for Clean Energy, Dalian Institute of Chemical Physics, Chinese Academy of Sciences Dalian 116023 China canli@dicp.ac.cn

## Abstract

Low-concentration CO_2_ capture is particularly challenging because it requires highly selective adsorbents that can effectively capture CO_2_ from gas mixtures containing other components such as nitrogen and water vapor. In this study, we have successfully developed a series of controlled alkali-etched MOF-808-*X* (where *X* ranges from 0.04 to 0.10), the FT-IR and XPS characterizations revealed the presence of hydroxyl groups (–OH) on the zirconium clusters. Low-concentration CO_2_ capture experiments demonstrated improved CO_2_ capture performance of the MOF-808-*X* series compared to the pristine MOF-808 under dry conditions (400 ppm CO_2_). Among them, MOF-808-0.07 with abundant Zr–OH sites showed the highest CO_2_ capture capacity of 0.21 mmol g^−1^ under dry conditions, which is 70 times higher than that of pristine MOF-808. Additionally, MOF-808-0.07 exhibited fast adsorption kinetics, stable CO_2_ capture under humid air conditions (with a relative humidity of 30%), and stable regeneration even after 50 cycles of adsorption and desorption. *In situ* DRIFTS and ^13^C CP-MAS ssNMR characterizations revealed that the enhanced low-concentration CO_2_ capture is attributed to the formation of a stable six-membered ring structure through the interaction of intramolecular hydrogen bonds between neighboring Zr–OH sites *via* a chemisorption mechanism.

## Abstract 2

We demonstrated that controlled alkali etching of MOF-808 with enhanced low-concentration CO_2_ capture capacity under dry conditions.

## Graphical abstract

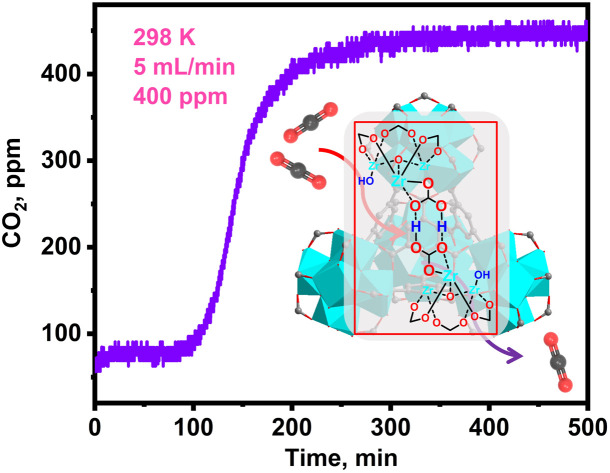

## Introduction

Direct air capture (DAC) of CO_2_ has emerged as a promising carbon negative approach to achieving carbon neutrality.^1–5^ However, the extremely low concentration of CO_2_ in the air (∼410 ppm) presents a significant challenge. Various solid adsorption materials such as zeolite, activated carbon, porous silicon, coordination polymers, metal–organic frameworks (MOFs), and covalent organic frameworks (COFs) have been explored for CO_2_ capture.^6–20^ MOFs, with their diverse structures and post-modification functionalization,^21^ show potential for CO_2_ adsorption, but are still challenging for low-concentration CO_2_ capture, especially for DAC. Currently, only a few ultra microporous MOFs and bioinspired MOFs are capable of capturing ultra-low concentrations of CO_2_.^22–31^ One of the most widely studied strategies for achieving low-concentration CO_2_ adsorption is amine modification, which has ultra-strong affinity for CO_2_ molecules.^32–37^ However, amine adsorption suffers from low adsorption kinetics, low amine efficiency and loss of amines, limiting its practical application.^38,39^ Therefore, the development of non-amine modified low-concentration CO_2_ adsorption MOFs-based materials is necessary. Zr-based MOFs with secondary building units (SBUs) show promise due to their SBUs and high coordination numbers.^40^ Controlled etching of these MOFs exposes more M–OH sites, and the microporous environment of MOFs enhances local CO_2_ enrichment and capture, but exploration in this area is still limited.

In this study, we synthesized a series of controlled alkali-etched MOF-808-*X* (*X*: 0.04–0.10) materials with enhanced low-concentration CO_2_ capture capacity under simulated air conditions compared to the pristine MOF-808. Among these materials, MOF-808-0.07 exhibited a CO_2_ capture capacity of 0.21 mmol g^−1^ under simulated air conditions, which is 70 times higher than that of the pristine MOF-808. Additionally, MOF-808-0.07 displayed excellent stability over 50 cycles of adsorption and desorption. *In situ* DRIFTS and ^13^C CP-MAS ssNMR analysis revealed that the increased low-concentration CO_2_ capture capacity is attributed to the formation of a stable six-membered ring structure through the interaction of intramolecular hydrogen bonds between neighbouring Zr–OH sites in the micro-mesoporous environment of MOF-808-*X*.

## Results and discussion

**Scheme 1:**
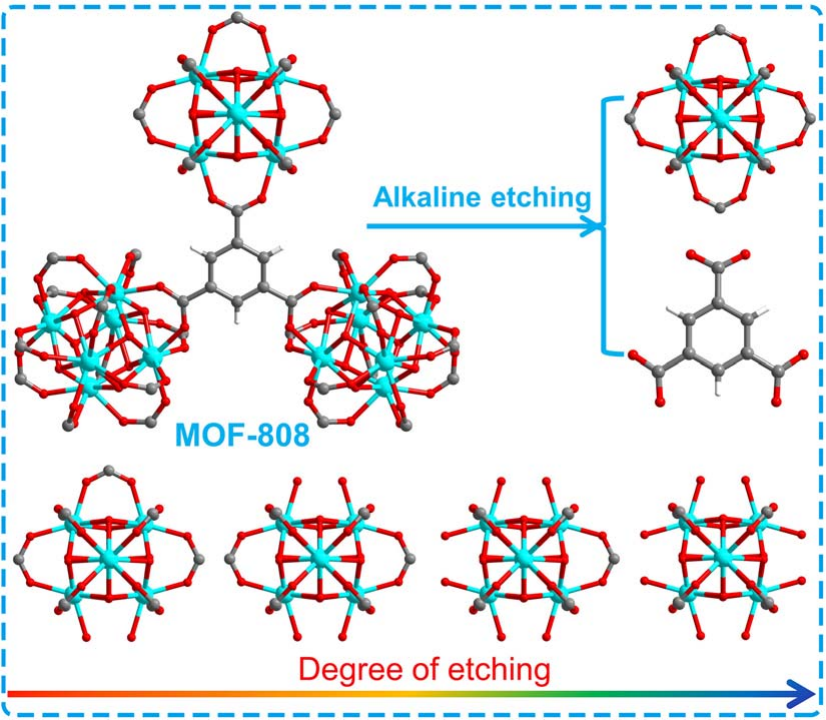
Schematic diagram of degree of etching for MOF-808 and series of MOF-808-*X* (*X*: 0.04–0.10).

**Fig. 1:**
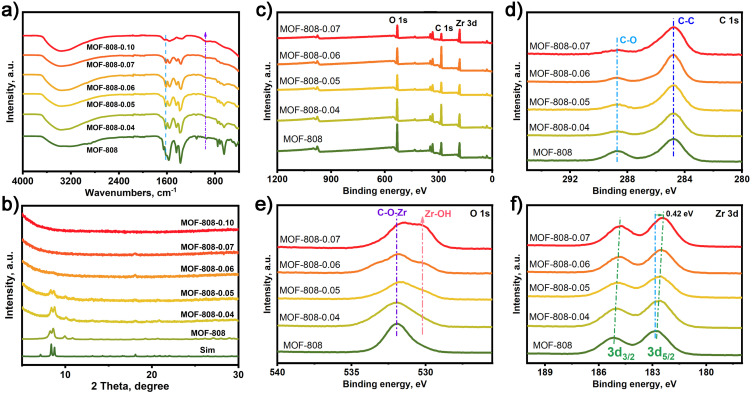
(a) FT-IR spectra, (b) PXRD patterns and (c–f) XPS survey, and high-resolution XPS spectra of the C 1s, O 1s, and Zr 3d of MOF-808 and MOF-808-*X* (*X*: 0.04–0.1).

MOF-808 was synthesized according to the reported method.^40,41^ And MOF-808-*X* (*X*: 0.04–0.10) series were prepared by various degrees of alkali etching of MOF-808 (Scheme 1). In Fig. 1a, the FT-IR analysis of these samples reveals that the infrared absorption peaks at 1630 and 1400–1600 cm^−1^, which correspond to the stretching vibration peak of C=O and the benzene ring, respectively, display varying degrees of weakening. This suggests that the benzene ring in MOF-808 has undergone degradation to different extents. In Fig. 1b, the powder X-ray diffraction (PXRD) patterns of the as-synthesized MOF-808 are shown, which match well with the simulated PXRD pattern obtained from single crystal analysis.^40^ However, the PXRD peaks of the MOF-808-*X* (*X*: 0.04–0.10) series gradually weaken with increasing etching degree, until all XRD diffraction peaks disappear. Scanning electron microscopy (SEM) images of the as-synthesized MOF-808 exhibit octahedral morphology (Fig. S1a†), consistent with previous literature reports. The alkali-etched MOF-808-*X* (*X*: 0.04–0.10) series show almost the same morphology as MOF-808 with varying degrees of etching (Fig. S1b–f†).

X-ray photoelectron spectroscopy (XPS) analyses were conducted to investigate the electronic structure of MOF-808 and MOF-808-*X* (*X*: 0.04–0.07) series (Fig. 1c). In Fig. 1d, the C 1s high-resolution spectrum of MOF-808 and MOF-808-*X* series displays two distinct binding energy peaks at 284.8 and 288.5 eV, corresponding to the binding energy peaks of C–C and C–O. The O 1s high-resolution spectrum of MOF-808 in Fig. 1e shows a binding energy peak of C–O–Zr bond at 532.5 eV. However, a new binding energy peak appeared in the O 1s HR-XPS spectrum at 530.5 eV, which gradually increased with the increase of the alkali etching degree of MOF-808, and the new binding energy peak was attributed to the Zr–OH generated by alkali etching. Moreover, it is obvious from Fig. 1f that the binding energy peak of Zr 3d is shifted towards a lower binding energy in MOF-808-*X* series compared to the pristine MOF-808. These results suggest that electron-donating groups exist on the Zr site.

N_2_ adsorption and desorption isotherms were employed to further characterize the pore structure and BET surface area of MOF-808 and MOF-808-*X* series. The isotherms of these materials exhibit a typical type I adsorption pattern (as shown in Fig. S2†), indicating the presence of micro-mesoporous structure. The BET specific surface area of MOF-808 was found to be 1614 m^2^ g^−1^, whereas for MOF-808-*X* (*X*: 0.04–0.07) series, the BET specific surface area gradually decreases with the increase in the degree of etching and is found to be 300, 229, 225, 221, and 144 m^2^ g^−1^, respectively. This suggests that the BET surface area changes as the degree of etching increases due to the gradual collapse of the MOF-808 framework.

**Fig. 2:**
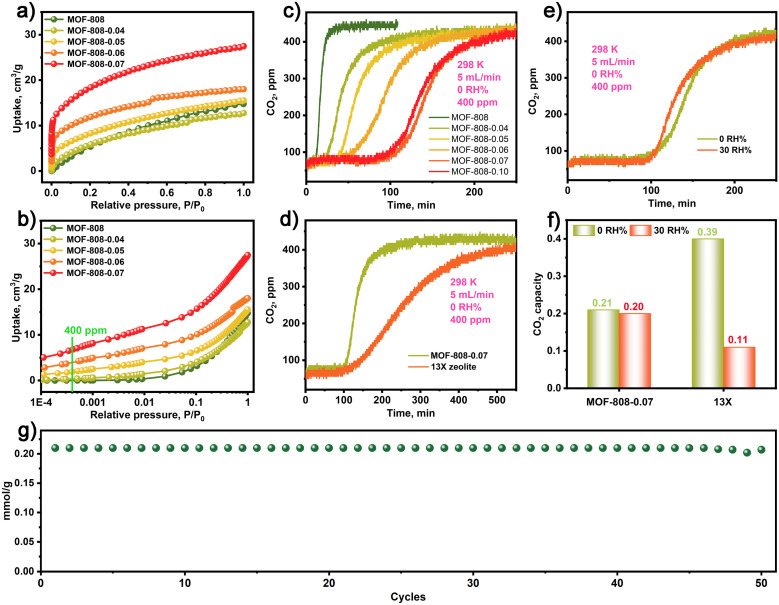
(a) and (b) CO_2_ adsorption isotherm for MOF-808 and MOF-808-*X* (*X*: 0.04–0.07) series. The dynamic CO_2_ breakthrough curves for MOF-808 and MOF-808-*X* (*X*: 0.04–0.07) series (c) and 13X zeolite under dry conditions (d). (e) The dynamic CO_2_ breakthrough curves for MOF-808-0.07 at 0 and 30 RH%. (f) Comparison of CO_2_ capture capacity for MOF-808-0.07 and 13X zeolite at 0 and 30 RH%, respectively. (g) The cycling stability of CO_2_ capture for MOF-808-0.07.

Due to the presence of numerous Zr–OH sites in the MOF-808-*X* series, we were prompted to investigate the CO_2_ adsorption characteristics of these materials. As shown in Fig. 2a, the CO_2_ adsorption isotherms of the MOF-808-*X* series demonstrate improved CO_2_ adsorption at low pressures compared to the pristine MOF-808. Particularly, the MOF-808-*X* series materials with Zr–OH sites exhibit a strong affinity for CO_2_ at low concentrations, as evidenced by the steepness of the CO_2_ adsorption isotherms and the attainment of a plateau at very low pressures. Further analysis of the CO_2_ adsorption behaviour (Fig. 2b) within a low-pressure range of 400 ppm reveals that MOF-808-0.07 exhibits a high CO_2_ uptake of 0.28 mmol g^−1^, which is comparable to the values obtained for MOF-808-0.04 (0.01 mmol g^−1^), MOF-808-0.05 (0.08 mmol g^−1^), MOF-808-0.06 (0.16 mmol g^−1^), and the pristine MOF-808 (0.008 mmol g^−1^). This highlights the significantly enhanced CO_2_ uptake and the interactions between CO_2_ and Zr–OH sites in the MOF-808-*X* series materials compared to the pristine MOF-808. Additionally, the MOF-808-*X* series exhibits excellent thermal stability up to 200 °C (Fig. S3†).

The dynamic CO_2_ capture performance of MOF-808 and MOF-808-*X* series were assessed in a fixed-bed reactor packed with a column of simulated ambient air (400 ppm CO_2_ and argon as balance gas) under flow conditions (5 mL min^−1^) at 298 K. The detailed experimental procedure is provided in the ESI.†Fig. 2c depicts the short-term CO_2_ breakthrough process of pristine MOF-808 in simulated dry air conditions (0 RH%), resulting in low CO_2_ capture capacities of 0.003 mmol g^−1^. In contrast, MOF-808-*X* (*X*: 0.04–0.10) series exhibited long-term dynamic CO_2_ breakthrough processes with enhanced CO_2_ capture capacity compared to the pristine MOF-808. The dynamic CO_2_ capture capacity of MOF-808-*X* (*X*: 0.04–0.10) series under simulated dry air conditions were 0.06, 0.09, 0.13, 0.21, and 0.205 mmol g^−1^, respectively. Notably, the MOF-808-0.07 demonstrated the highest CO_2_ capture capacity, which is a 70-fold increase in CO_2_ uptake capacity compared to the pristine MOF-808. Although the MOF-808-*X* series exhibited lower CO_2_ capture capacity than the 13X zeolite (0.39 mmol g^−1^) under simulated dry air conditions, they demonstrated faster adsorption kinetics than 13X zeolite, as illustrated by the sharper breakthrough profile for MOF-808-0.07 compared to 13X (Fig. 2e). Additionally, Fig. 2d indicates that MOF-808-0.07 exhibited almost the same CO_2_ breakthrough curves under simulated dry and humid air conditions (0 and 30 RH%). In contrast, the 13X zeolite in Fig. 2f exhibited significantly reduced CO_2_ capture capacity under humid air conditions (30 RH%), indicating that MOF-808 has higher moisture resistance. Moreover, Fig. 2g demonstrates that MOF-808-0.07 exhibited stable CO_2_ capture performance with minimal losses after 50 cycles (Fig. S4†). In addition, the MOF-808-0.07 after CO_2_ capture was evaluated by FT-IR, PXRD, SEM, XPS and N_2_ adsorption and desorption isotherms, all results show the structure integrity for MOF-808-0.07 in CO_2_ capture processing (Fig. S5–S9†). The above results indicate that the MOF-808-0.07 has superior CO_2_ adsorption–desorption stability.

In order to illustrate the low-concentration CO_2_ adsorption process of MOFs containing Zr-SBUs at low concentrations, we synthesized a series of MOFs with different M-SBUs, including MIL-101-Fe with Fe_3_-SBU cluster, MIL-101-Cr with Cr_3_-SBU cluster, and MIL-125-Ti with Ti_4_-SBU cluster. Through controlled etching, as confirmed by PXRD analysis (Fig. S10†), we obtained MOFs with varying degrees of etching. The dynamic CO_2_ capture results revealed that all MOFs with varying degrees of etching exhibited a CO_2_ capture process, but their capture capacities were not comparable to that of MOF-808-*X* with controlled etching with Zr_6_-SBU cluster (Fig. S11†). This suggests that MOFs with higher coordination numbers exhibit superior CO_2_ capture abilities.

**Fig. 3:**
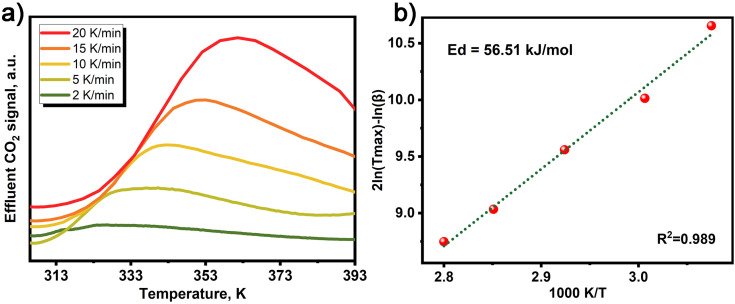
(a) TPD results for CO_2_ desorption from MOF-808-0.07 in dry conditions after being saturated with CO_2_ from a gas stream of 400 ppm CO_2_. (b) Microkinetic analysis assuming first order desorption.

To investigate the desorption kinetics of MOF-808-0.07 in dry air conditions, we employed temperature programmed desorption (TPD) to evaluate its desorption energy. The activation energies of desorption for MOF-808-0.07 were calculated using the method proposed by Cvetanovic and Amenomiya, by measuring the TPD-CO_2_ signal at different heating rates, as presented in Fig. 3a and b.^42^ Our results demonstrate that MOF-808-0.07 exhibits a higher desorption energy (56.51 kJ mol^−1^) than 13X zeolite (48.14 kJ mol^−1^ (ref. 42)) under simulated dry air conditions (Table S1†), indicating that CO_2_ adsorption by MOF-808-0.07 occurs through chemical adsorption.

**Fig. 4:**
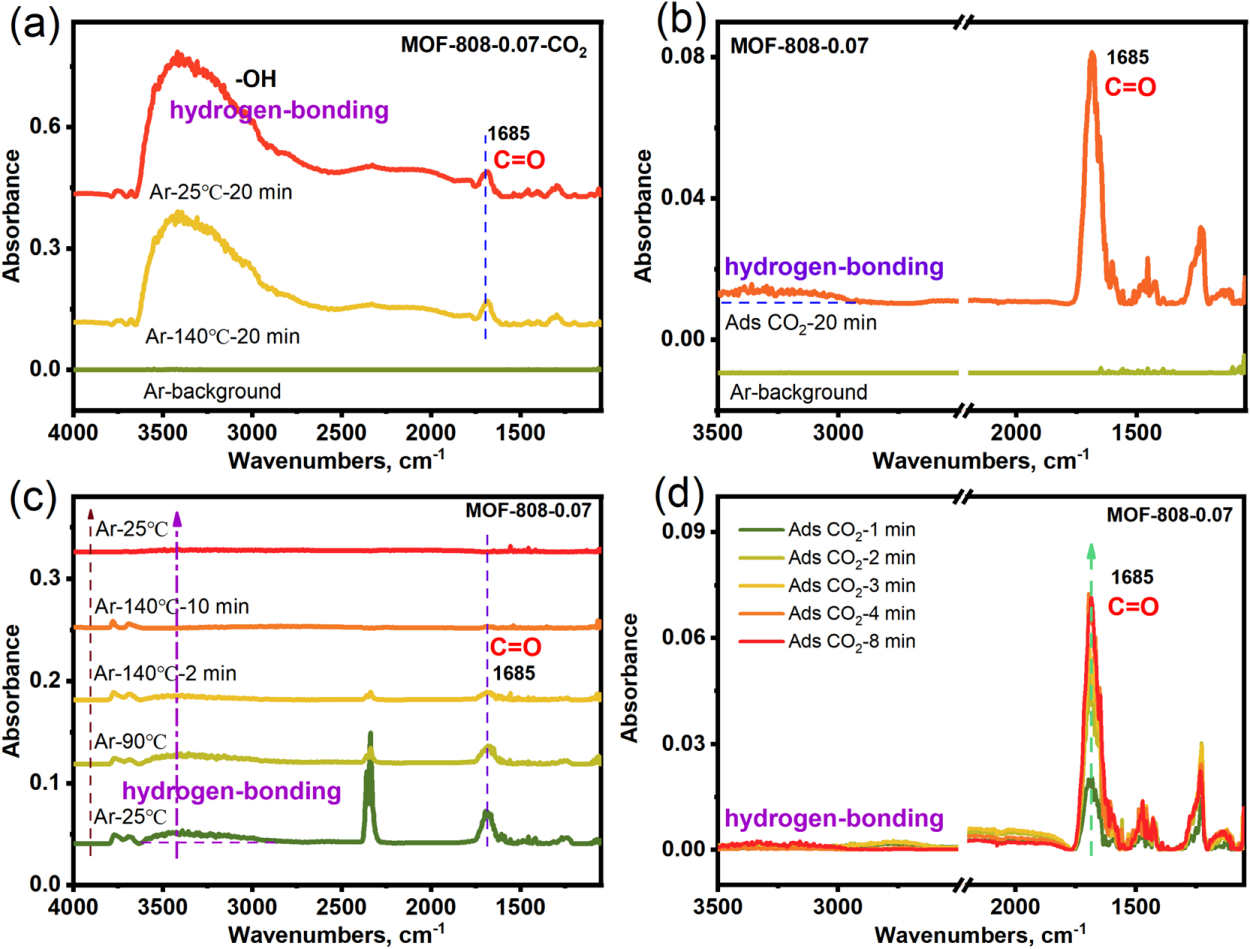
*In situ* DRIFTS of (a) MOF-808-0.07-CO_2_ for desorption CO_2_, (b) MOF-808-0.07 for adsorption CO_2_, (c) MOF-808-0.07-CO_2_ for the 2nd desorption CO_2_, (d) MOF-808-0.07 for the 2nd adsorption CO_2_.

In order to verify the adsorbed species in CO_2_ capture for MOF-808-0.07, the *in situ* diffuse reflectance infrared Fourier transform spectroscopy (*in situ* DRIFTS) of MOF-808-0.07 with adsorbing CO_2_ in simulated dry air (MOF-808-0.07-CO_2_) was carried out. Fig. 4a shows two distinct infrared absorption peaks at 1685 and 3000–3600 cm^−1^ in the *in situ* DRIFTS spectra of MOF-808-0.07-CO_2_ after heat treatment (140 °C), corresponding to the stretching vibration peak of C=O (–OCO_2_H), and –OH (M-OH with broad peak and hydrogen-bonding), respectively. The results display that the CO_2_ adsorption within the MOF-808-0.07 framework is in the form of bicarbonate species and hydrogen bonding interactions under dry conditions. Furthermore, the heat-treated MOF-808-0.07 is subjected to *in situ* CO_2_ adsorption again in dry conditions, the *in situ* DRIFTS spectra in Fig. 4b show obvious infrared absorption peaks in 1685 and 3000–3500 cm^−1^ corresponding to the stretching vibration peak of C=O (–OCO_2_H) and hydrogen-bonding. After heat treatment again, the infrared absorption peak of C=O and hydrogen-bonding gradually disappeared again (Fig. 4c), demonstration of the breaking of hydrogen bonding and the successful complete desorption of CO_2_. Further elucidating the adsorption–desorption stability, the second *in situ* CO_2_ adsorption also showed that the C=O and hydrogen-bonding infrared absorption peak gradually strengthens with various adsorption time (Fig. 4d). As a comparison, the control experiments of pristine MOF-808-CO_2_ shows no obvious infrared absorption peak for CO_2_ desorption at 140 °C. And the heat-treated MOF-808 is subjected to *in situ* CO_2_ adsorption again in dry conditions along with various time, the *in situ* DRIFTS spectra show no obvious change in infrared absorption peaks (Fig. S12†). The results show that parent MOF-808 does not have low concentration CO_2_ adsorption capacity. Based on the above *in situ* DRIFTS results, showing that the alkali etched MOF-808-0.07 has enhanced low concentration CO_2_ capture capacity compared to parent MOF-808 under dry air conditions due to the presence of Zr–OH adsorption sites.

**Fig. 5:**
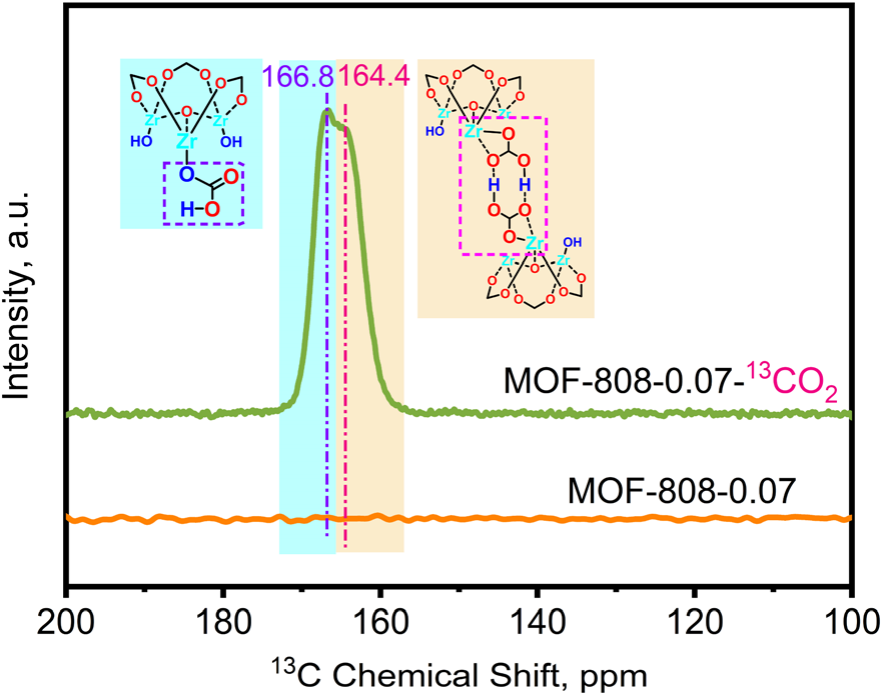
Stacked plots of solid-state ^13^C CP-MAS NMR spectra of MOF-808-0.07 before and after adsorption of ^13^CO_2_.

To elucidate the formation of –OCO_2_H species under dry conditions, solid-state cross-polarization magic-angle spinning (CP-MAS) ^13^C NMR experiments were conducted on variant MOF-808-0.07 to investigate the change in chemical species before and after capturing ^13^CO_2_ (isotopic gas). Fig. 5 displays the ^13^C CP-MAS ssNMR spectrum of the pristine MOF-808 without adsorbed CO_2_, showing no observable chemical shifts, indicating complete etching of the carbon framework in MOF-808. Upon adsorption of ^13^CO_2_ under dry conditions, two distinct chemical shifts appeared in the ^13^C CP-MAS ssNMR spectrum at *δ*^13^C = 166.8 and 164.4 ppm. Combining these results with the *in situ* DRIFTS data, it can be inferred that these shifts are attributed to –OCO_2_H groups and –OCO_2_H groups involved in intramolecular hydrogen bonding, respectively.

**Fig. 6:**
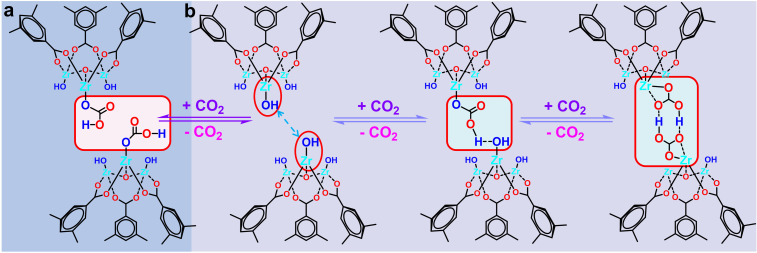
Proposed low-concentration CO_2_ capture mechanism for MOF-808-*X* series.

Based on the above *in situ* DRIFTS and ^13^C CP-MAS ssNMR characterizations, we proposed a possible mechanistic of low-concentration CO_2_ capture process in MOF-808 series. (1) When the two Zr–OH sites within the MOF-808-*X* framework are distanced apart, each Zr–OH site can adsorb one CO_2_ molecule, forming Zr–O_2_COH species (Fig. 6a). (2) When the neighbouring Zr–OH sites within the MOF-808-*X* framework are in close proximity. As shown in Fig. 6b, first, a Zr–OH site adsorbs a CO_2_ molecule to form a Zr–O_2_COH species, and the Zr–O_2_COH species forms intramolecular hydrogen bonding with the neighbouring Zr–OH site. Subsequently, the neighbouring Zr–OH re-adsorbs a CO_2_ molecule with it to form two opposing Zr–O_2_COH species, which interact to form a stable six-membered ring structure through the interaction of intramolecular hydrogen bonding to complete an adsorption process.

## Conclusions

In conclusion, we have demonstrated that controlled alkali etching of MOF-808 leads to the formation of MOF-808-*X* series, which exhibit significantly enhanced low-concentration CO_2_ capture compared to the pristine MOF-808 under dry air conditions. Among the MOF-808-*X* series, MOF-808-0.07 displays the highest CO_2_ capture capacity of 0.21 mmol g^−1^ in simulated dry air conditions, which is 70 times higher than the pristine MOF-808. The desorption kinetics of the MOF-808-0.07 also show higher desorption energy compared to the commonly used 13X zeolite. Our control experiments suggest that MOFs with high coordination numbers show higher CO_2_ capture performance under dry air conditions. Furthermore, *in situ* DRIFTS and ^13^C CP-MAS ssNMR results indicate that the enhanced low-concentration CO_2_ capture is due to the formation of a stable six-membered ring structure through intramolecular hydrogen bonds between Zr–OH sites of neighbouring micro-mesoporous environments of MOF-808-*X*. Overall, these findings suggest the potential of MOF-808-*X* series as promising materials for low-concentration CO_2_ capture.

## Data availability

The authors declare that all data supporting the findings of this study are available from the corresponding author upon reasonable request.

## Author contributions

Hong Dong: conceptualization, data curation, formal analysis, investigation, funding acquisition, writing – original draft, writing – review & editing. Lihua Li: investigation and formal analysis, Can Li: supervision, funding acquisition, project administration and writing – review & editing.

## Conflicts of interest

There are no conflicts to declare.

## Supplementary Material

SC-014-D3SC03213B-s001
