# Supplementary material for: Controlled alkali etching of MOFs with secondary building units for low-concentration CO2 capture
Source: Chem Sci. 2023 Jul 15;14(32):8507–13. doi: 10.1039/d3sc03213b (PMC10430719; doi:10.1039/d3sc03213b)
Supplement: SC-014-D3SC03213B-s001 [file SC-014-D3SC03213B-s001.pdf]

Electronic Supplementary Material (ESI) for Chemical Science.
This journal is © The Royal Society of Chemistry 2023

Supplementary Information

Controlled alkali etching of MOFs with secondary building units for low-concentration CO_2 capture

Hong Dong, Lihua Li, and Can Li*

State Key Laboratory of Catalysis, Dalian Institute of Chemical Physics, Chinese Academy of Sciences, Dalian 116023 (China).

E-mail: canli@dicp.ac.cn

S1. Materials and reagents

All of the reagents and solvents were commercially available and used without further purification.

S2. Characterizations

Thermogravimetric analysis (TGA) was carried out on a Diamond TG/DTA analyzer at a heating rate of 10 °C/min from ambient temperature to 800 °C in N_2 atmosphere. Scanning electron microscopy (SEM) and EDS micrographs were recorded on a JSM-7800 instrument operating at 20 kV. X-ray powder diffraction (PXRD) patterns were recorded on a D/Max 2500 X-ray diffractometer. The patterns were collected at a scanning rate of 5° per min in the 2θ range from 5-30°. Nitrogen adsorption-desorption isotherms were measured at 77 K on a Micromeritics ASAP 2020 absorptiometer, and CO_2 adsorption isotherms were measured at 298 K. XPS was carried out with a Thermo Scientific. The *in-situ* DRIFTS experiments were performed in an IR cell made of quartz. Before the DRIFTS measurements were initiated, the sample was treated under N_2 flow (30 mL/min) for 30 min to ensure a clean and stable environment. After that, heat to 150 °C for 20 min, then the spectra data are collected in the range of 4000-800 cm^{-1} at regular intervals. The results are reported after subtraction the corresponding background registered under N_2 atmosphere. CO_2-TPD-MS was conducted by ASAP-2020, the samples were heated to 180 °C for 30 min, and then the spectra data are collected instantly.

S3. Synthesis of materials

Synthesis the MOF-808

The MOF-808 was synthesized by improving the synthesis method of the literature.[1] As follows: 3 mmol of $ZrOCl_2$ and 3 mmol 1,3,5-benzenetricarboxylic acid (BTC) were dissolved in 30 mL of DMF with 30 mL of HCOOH as a modulator. The mixed precursor solution was is stirred by reflux to obtain target products. After cooling to room temperature, the obtained white solid was washed three times with DMF and acetone soaked three times. Finally, vacuum dry for 12 hours.

Synthesis the MOF-808-X (0.04 - 0.10) series

The MOF-808-X (0.04-0.10) series were synthesized via various degree alkali etching strategy. As follows: A certain amount of MOF-808 was first dispersed in tetrahydrofuran solution, and then different amounts of NaOH were added. Stiring at room temperature for 24 hours to obtain a series of target products. The obtain products were denoted as MOF-808-X, where X presents the different mass of adding NaOH (X: 0.04, 0.05, 0.06, 0.07 and 0.10 represent the mass of adding NaOH, respectively).

S4. Dynamic CO_2 Breakthrough testing

The CO_2 dynamic breakthrough experiments were accomplished by a dynamic gas breakthrough set-up (Scheme S1). The experiment was conducted using a packed bed with column (6 mm inner diameter × 30 mm). The column packed with sample (0.05 g) was firstly activated with Ar flow (40 mL min^{-1}) for 1 h at 413 K. After activation and cooling down to the desired adsorption temperature, simulated air (CO_2/Ar) with 10 mL min^{-1} was introduced. Outlet gas from the column was monitored using a CO_2 analyzer (A-MTDS 990, Airmonic). After the breakthrough experiment, the column was regenerated with Ar flow (40 mL min^{-1}) at 413 K for 1 h. CO_2 capture capacity was calculated as follow:

$$q = \frac{1}{w}\left[\int_0^t Q \times \frac{C_{in} - C_{eff}}{1 - C_{eff}} dt\right]\frac{T_0}{T}\frac{1}{V_m}$$

W: the weight of adsorbent (mg); Q: the gas flow rate (mL/min); C_{in} and C_{eff}: CO_2 volume fractions at the influent and effluent of the reactor, respectively. T_0 is 273 K, T is the gas adsorption temperature (K), and V_m is standard gas volume (22.4 L/mol). For simulated air, the value of 1-C_{eff} was estimated to be 1 (the largest C_{eff} is of 400 ppm).

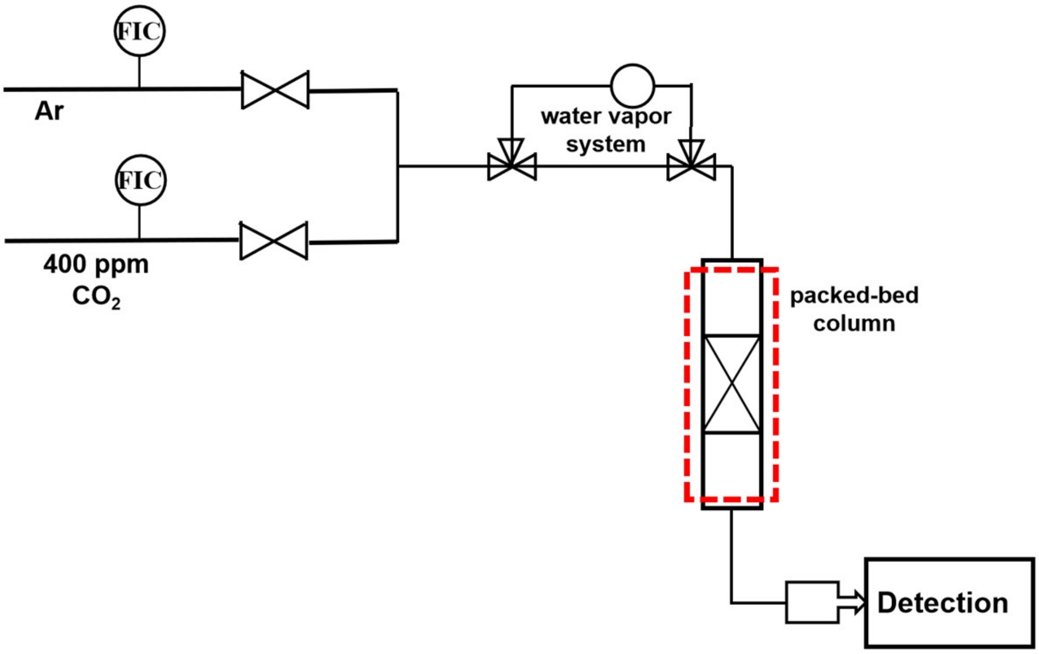

Scheme S1. Schematic diagram of the CO_2 capture device.

S5. Estimation of desorption kinetic parameters using temperature programmed desorption (TPD)

The TPD experiments were carried out in the same setup as in the column breakthrough measurements. MOFs were loaded in a quartz tubing. Before the TPD experiments, the samples were subjected to outgassing at 150°C for 2 hours under a flow of Ar gas at a rate of 40 mL/min. After the temperature was lowered to 25ºC, the samples were saturated with a gas stream of 400 ppm CO_2/ Ar at a flow rate of 10 $mL\cdot min^{-1}$. After saturation, TPD experiments were carried out by switching the gas stream to Ar at a flow rate of 20 $mL\cdot min^{-1}$ and heating up with a constant ramp rate (2, 5, 10, 15, 20 $°C\cdot min^{-1}$). Simultaneously, the signal of CO_2 was detected using a mass spectrometer with m/z= 44.

S6. Supplementary Figures

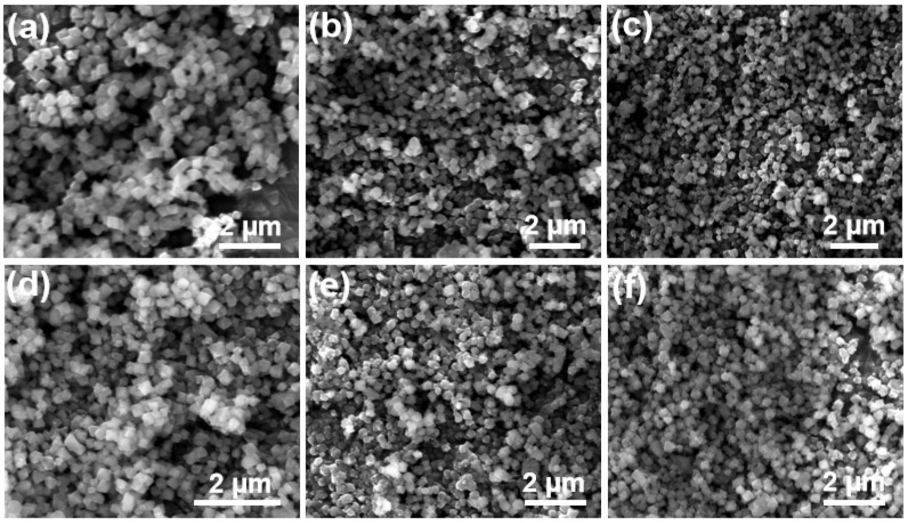

Figure S1. a-f) SEM images of MOF-808 and MOF-808 (0.04 - 0.10) series.

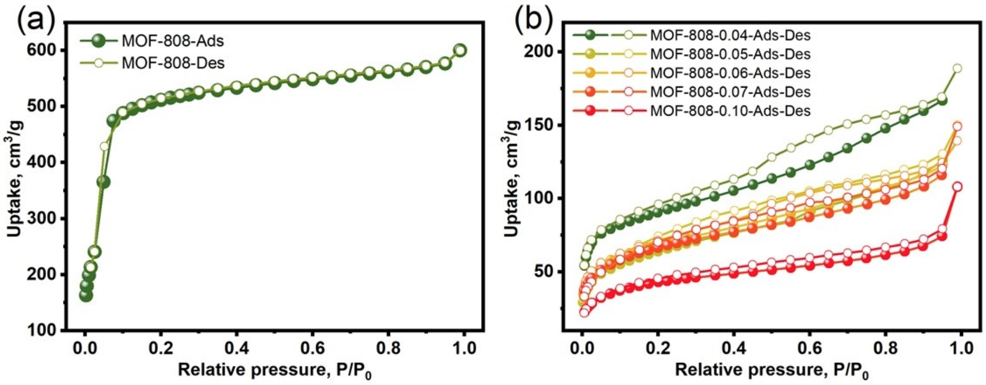

Figure S2. N_2 adsorption and desorption isotherms of, a) MOF-808, and b) MOF-808 (0.04 - 0.10) series.

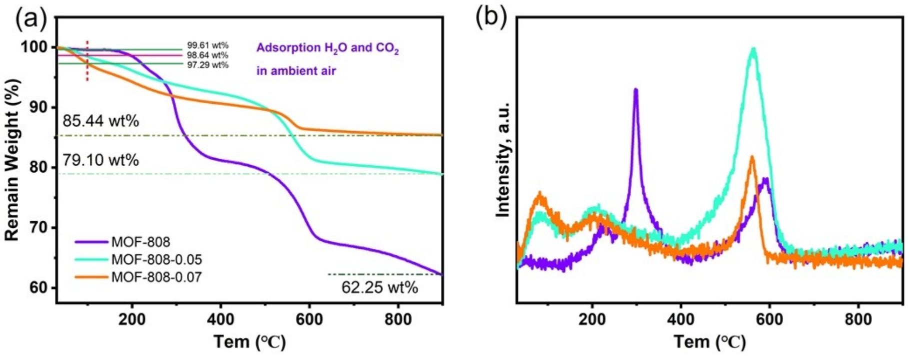

Figure S3. TG and DTG curves of MOF-808, MOF-808-0.05 and MOF-808-0.07.

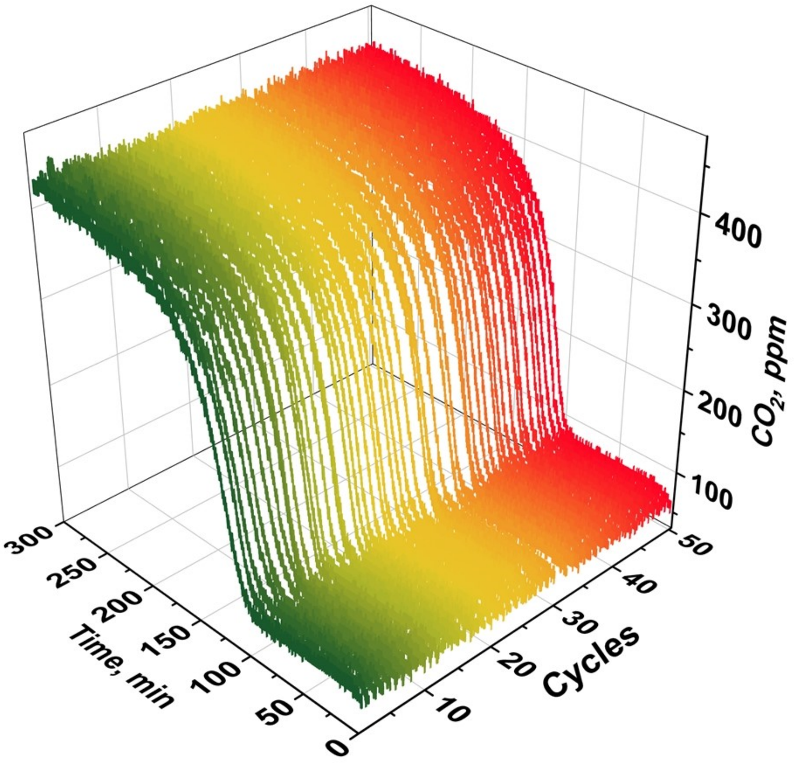

Figure S4. The cycling stability of CO_2 capture for MOF-808-0.07.

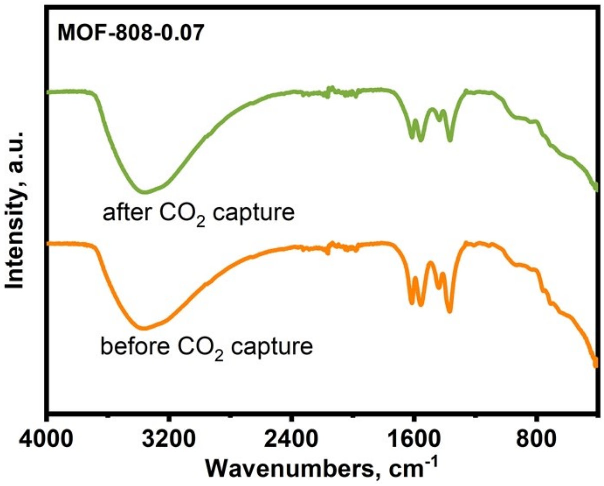

Figure S5. The FT-IR spectra of MOF-808-0.07 before and after CO_2 capture.

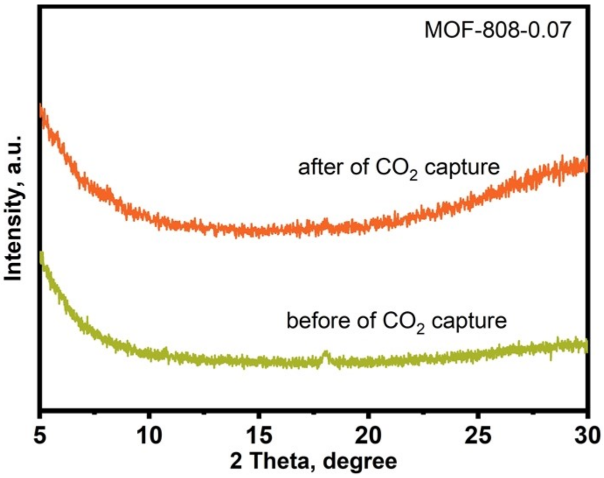

Figure S6. The PXRD patterns of MOF-808-0.07 before and after CO_2 capture.

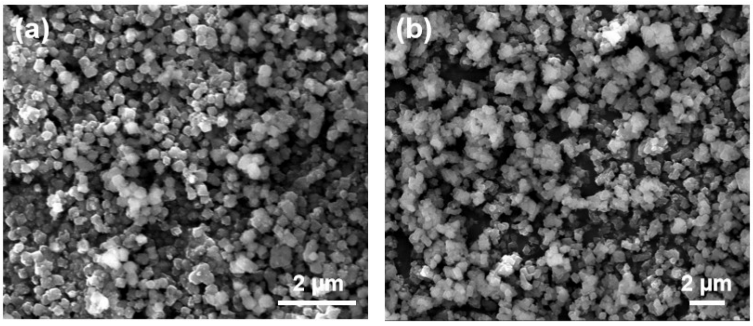

Figure S7. The SEM images of MOF-808-0.07 before and after CO_2 capture.

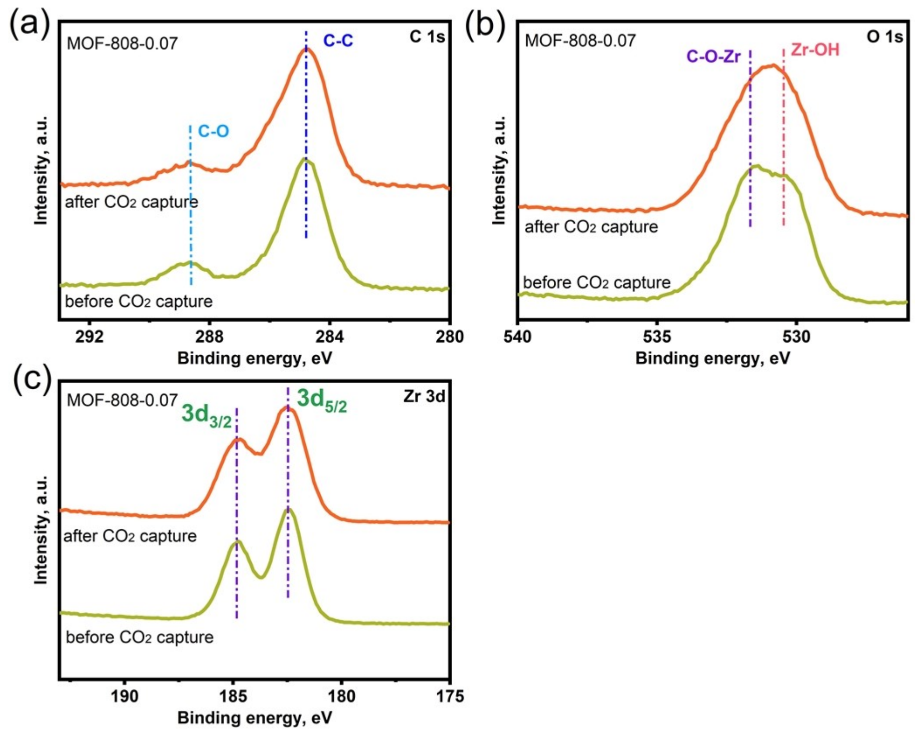

Figure S8. high-resolution XPS spectra of the C 1s, O 1s, and Zr 3d of MOF-808-0.07 before and after CO_2 capture.

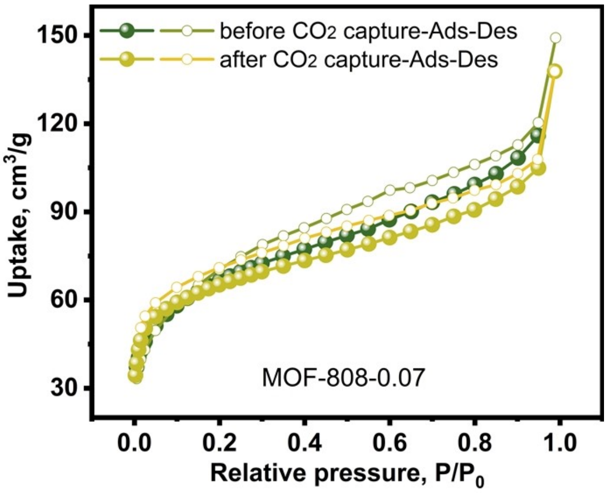

Figure S9. The N_2 adsorption and desorption isotherms of MOF-808-0.07 before and after CO_2 capture.

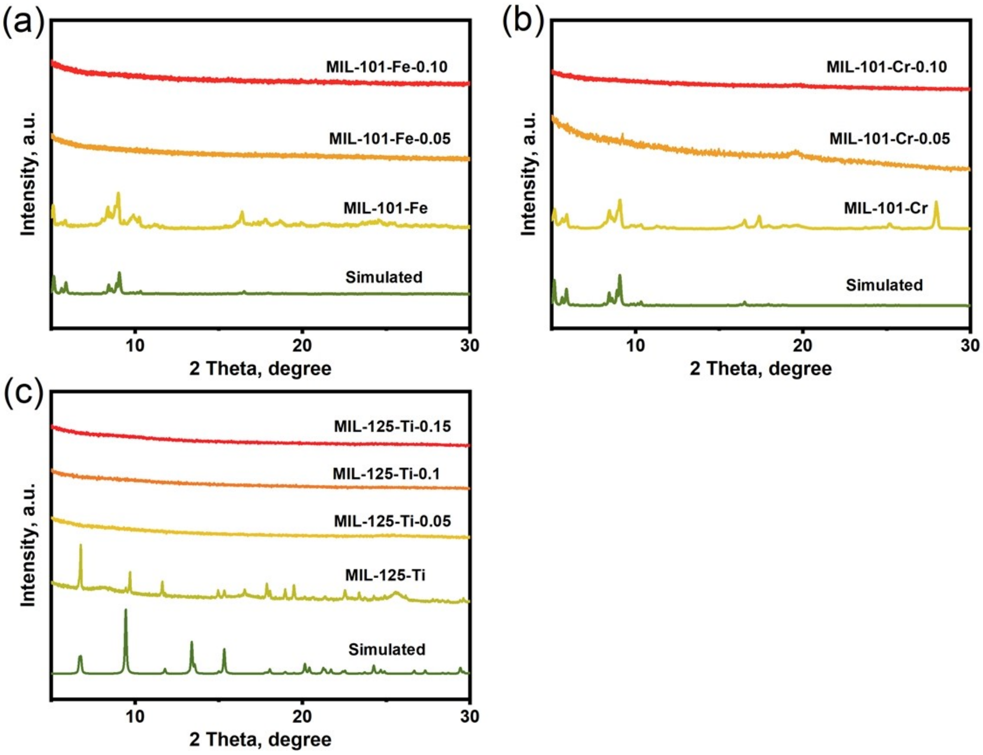

Figure S10. PXRD patterns of MIL-101-Fe series (a), MIL-101-Cr series (b), MIL-125-Ti series (c).

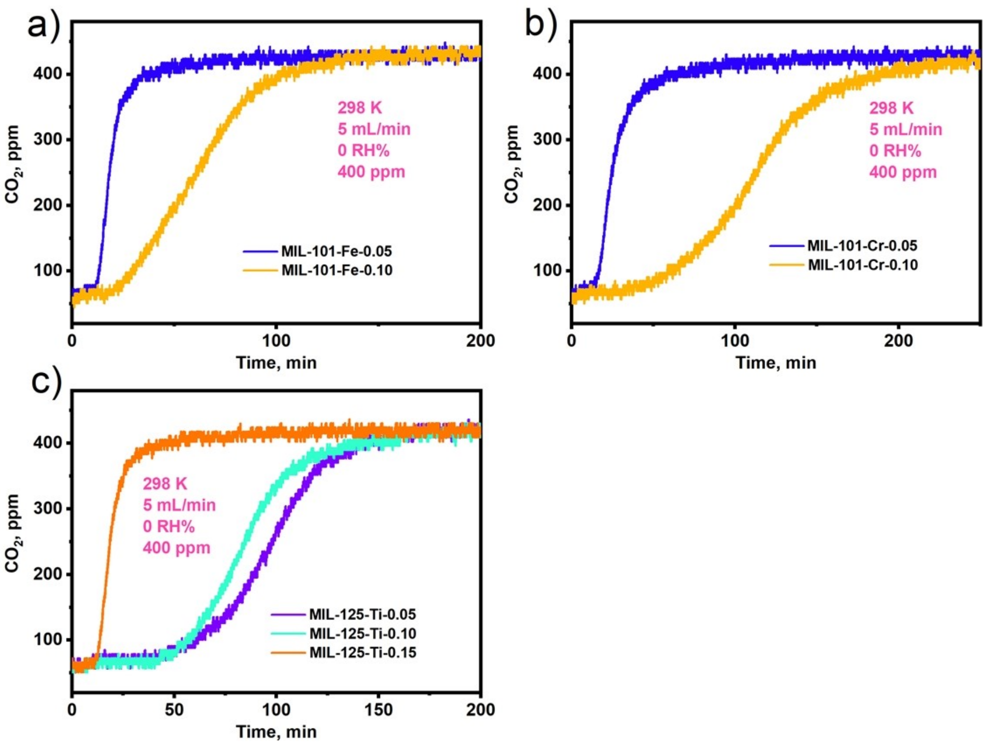

Figure S11. The dynamic CO_2 breakthrough curves of MIL-101-Fe series (a), MIL-101-Cr series (b), and MIL-125-Ti series (c).

S7. Kinetics for desorption using temperature programmed desorption

Desorption kinetic parameters of CO_2 from MOF-808-0.07 in simulated dry conditions was estimated using temperature programmed desorption (TPD). The method developed by Cvetanovic and Amenomiya was applied with assumption of 1 st order desorption and homogeneous adsorption surfaces.[2] Generally, a linear relationship between $2\ln(T_m) - \ln\beta$ and $1/T_m$ can be established (see equation 1).

$2ln(T_m) - ln\beta = E_d/RTm + ln(E_d/AR)$ (equation 1)

Where T_m is the temperature of peak maximum (in K), β is the constant heating rate (in $K \cdot s^{-1}$), E_d is the activation energy for desorption, A is pre-exponential factor for desorption, R is universal gas constant (8.314 $J \cdot K^{-1} \cdot mol^{-1}$)

Therefore, the activation energy (E_d) and pre-exponential factor (A) for desorption can be obtained from the slope and intercept of a plot $2\ln(T_m) - \ln\beta = f(1/T_m)$, respectively. Please note that the activation energy (E_d) for desorption obtained is contributed from the intrinsic activation energy for desorption, diffusion and readsorption.[3] In particular for porous materials like zeolites, where diffusion and readsorption from the micropores are inevitable. [4]

Table S1. Kinetic parameters obtained from TPD analysis of CO_2 desorption from MOF-808-0.07 in simulated dry conditions.

Samples			**T_m (K)**			Ed (kJ/mol)
2-UIO-EDA	321	332	340	352	363	56.51
13X zeolite						48.14[5]

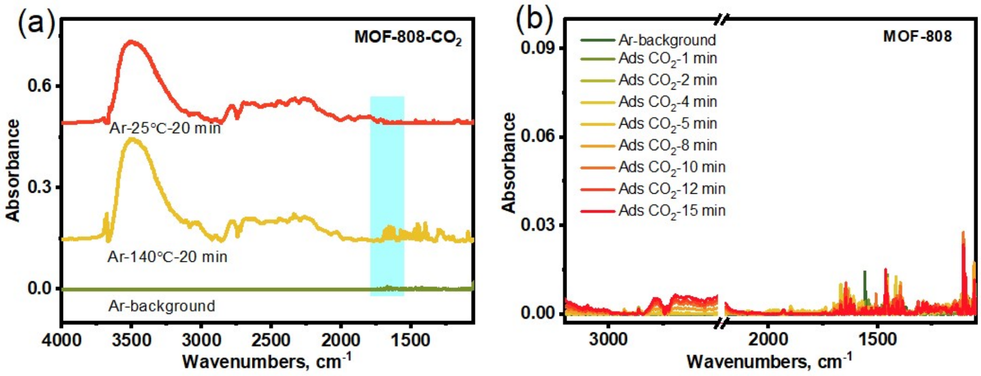

Figure S12. *In-situ* DRIFTS of a) MOF-808-CO_2 for desorption CO_2, b) MOF-808 for adsorption CO_2.

References

(1) Dong, H.; Yang, G.; Zhang, X.; Meng, X.; Sheng, J.; Sun, X.; Feng, Y.; Zhang, F. *Chem. - Eur. J.* **2018,** *24* (64), 17148-17154.

(2) R. J. Cvetanović, Y. Amenomiya, in *Adv. Catal.* (Eds.: D.D. Eley, H. Pines, P.B. Weisz), Academic Press, **1967**, pp. 103–149.

(3) R. E. Richards, L. V. C. Rees, *Zeolites* **1986**, 6, 17–25.

(4) X. Xia, J. Strunk, S. Litvinov, M. Muhler, *J. Phys. Chem. C* **2007**, 111, 6000–6008.

(5) Fu, D. L.; Park, Y.; Davis, M. E. Zinc Containing Small-Pore Zeolites for Capture of Low Concentration Carbon Dioxide. *Angew. Chem., Int. Ed*. **2022**, 61, e202112916.
